# Low Dose Contrast Enhanced CT Thorax Protocol: Comparison of Low Kilovoltage, Low Contrast Volume Using Iterative Reconstruction Technique with Standard Protocol

**DOI:** 10.4314/ejhs.v35i2.2

**Published:** 2025-03

**Authors:** Cauvery Sirdeshpande, Karthikeya D Hebbar, Saikiran Pendem

**Affiliations:** 1 Department of Medical Imaging Technology, Manipal College of Health Professions, Manipal Academy of Higher Education, Karnataka, Manipal, 576104, India; 2 Department of Radiodiagnosis and Imaging, Kasturba Medical College, Manipal Academy of Higher Education, Karnataka, Manipal, 576104, India

**Keywords:** Computed tomography, Radiation dose, Iterative reconstruction, Low dose, Contrast-to-noise ratio

## Abstract

**Background:**

Computed enhanced computed tomography (CECT) of the thorax is an effective imaging technique for diagnosing lung diseases. However, the increased use of CECT thorax scans has raised concerns regarding cancer risk and contrast-induced nephropathy (CIN). The iterative reconstruction (IR) method, specifically iDose4, enhances image quality (IQ) and reduces artifacts at low doses (LD). This study aimed to evaluate the image quality (IQ) and radiation dose (RD) of low-dose, low-volume (LD-LV) CECT thorax with iDose4, compared to standard dose (SD) CECT thorax (iDose4).

**Methods:**

Group A consisted of 40 patients who underwent SD CECT thorax (120 kVp, 60 ml), while Group B included 40 patients who underwent LD-LV CECT thorax (80 kVp, 40 ml). All CECT thorax scans were performed using a 128-slice Incisive CT (Philips Healthcare Systems). A qualitative analysis of thoracic structures in both the lung and mediastinal windows was performed. Quantitative parameters, including Hounsfield units (HU) for the pulmonary artery (CTPA) and infraspinatus muscle (CTISM), noise (SD), and contrast-to-noise ratio (CNR), were also assessed. The Mann-Whitney U test and independent t-test were used to compare IQ and radiation dose between the two groups.

**Results:**

Qualitative analysis of thoracic structures in the lung and mediastinal windows revealed no significant difference (p > 0.001) between the two groups. Quantitative parameters, such as CTPA, CTISM, and CNR, showed statistically significant differences (p < 0.001), with higher values observed in the LD-LV group compared to the SD group. The effective dose (ED) was reduced by 65.2% in the LD-LV group.

**Conclusion:**

Our LD-LV CECT thorax protocol using iDose4 demonstrated a significant reduction in effective dose and iodine contrast volume, while maintaining image quality and enhancing diagnostic confidence.

## Introduction

Computed tomography (CT) has revolutionized modern diagnostic imaging and decision-making processes since its introduction in 1970 (1). Chest CT is a widely used modality for detecting lung diseases, and the early detection of lung cancer through Chest CT has led to a 20% reduction in its mortality rate. Contrast-enhanced CT (CECT) thorax is particularly useful for identifying the blood supply to pulmonary nodules, aiding in the early detection and diagnosis of lung cancer (2-4). Globally, an estimated 375 million CT procedures are performed annually, with a growth rate of 3-4% per year. The global CT market has experienced significant changes due to the evolving needs of physicians and other healthcare professionals, as well as technological advancements (5). However, this has raised concerns about radiation exposure. In recent years, medical radiation exposure per person has increased due to the higher radiation levels associated with CT examinations, which are significantly more intense than conventional X-rays. For example, a thorax CT scan provides 100 times more radiation than a routine chest radiograph. Studies have shown that CT scans significantly raise the risk of cancer, and this risk is positively correlated with the CT dose (6-7).

To ensure diagnostic images are of high quality while minimizing radiation doses, dose optimization should follow the “As Low as Reasonably Achievable” (ALARA) principle. Reducing tube voltage (kVp) and tube current (mA) can decrease radiation doses but may also increase image noise (8-9). Filtered back projection (FBP) has been the traditional image reconstruction technique for 40 years; however, it is less effective in improving image quality at reduced kVp. In contrast, newer Iterative Reconstruction (IR) algorithms offer significantly improved noise performance at low radiation doses. The IR technique iteratively reconstructs images to more accurately estimate mathematical assumptions, thus providing images with reduced noise. The IR methods have made low-dose (LD) CT comparable to standard-dose (SD) CT in terms of diagnostic effectiveness and characterization capacity (10-11). iDose4, a fourth-generation reconstruction method developed by Philips HealthCare, improves image quality (IQ) and reduces artifacts at low doses. This algorithm “performs noise reduction in the projection and image data,” analyzing the projection data to identify noisy areas caused by low photon counts. Through iterative procedures, iDose4 preserves the gradients of underlying structures while minimizing noise in point measurements, ultimately enhancing IQ at low doses. Furthermore, the iDose4 algorithm reduces noise while retaining anatomical and pathological details (12-13).

Reducing kVp increases the photoelectric effect (PE) and helps reduce contrast volume, especially when combined with IR techniques. Patients with impaired kidney function are more susceptible to iodinated contrast-related acute kidney injury. A recent phantom study demonstrated that lower contrast media (CM) densities, along with lower tube potentials, result in superior contrast enhancement and maintained IQ in chest CT. The reduction in CM volume could provide significant benefits for patients with decreased renal function (14-15). Our literature review identified few studies that evaluated a low-dose (LD) low-volume (LV) contrast-enhanced CT (CECT) thorax protocol with reduced contrast volume compared to standard dose (SD). To our knowledge, this is the first study focused on using an LD protocol with 80 kVp and 40 ml of contrast for CECT thorax. Therefore, the current study aims to investigate the image quality (IQ) and radiation dose (RD) of LD-LV CECT (iDose4) thorax compared to SD (iDose4).

## Materials and Methods

This prospective study utilized historical controls. Approval was obtained from the Institutional Ethical Committee (IEC-506/2021). The study involved two groups. Group A consisted of 40 patients who underwent the standard-dose CECT Thorax protocol (120 kVp, 60 ml) between August 2020 and 2021. Since Group A included historical controls, informed consent (IC) was not obtained. Group B comprised 40 patients who underwent the low-dose, low-volume (LD-LV) CECT Thorax protocol (80 kVp, 40 ml) between September 2021 and February 2022. IC was obtained from all patients in Group B.

The inclusion criteria were patients with pulmonary nodules or masses and a BMI between 18.0 and 28.0 kg/m^2^. Limiting the BMI to this range ensures the study cohort represents individuals with a normal to slightly overweight body type, minimizing variations in image quality due to extreme body compositions. The exclusion criteria included patients with allergies to contrast media (CM), renal dysfunction, a BMI >28.0 or <18.0, or those who were pregnant. CECT Thorax scans were performed using a 128-slice Incisive CT (Philips Healthcare Systems, Cleveland, OH). The imaging parameters for Groups A and B are provided in Table 1. Iohexol (300 mgI/ml) was used as the contrast agent for both groups. A post-threshold (100 HU) delay of 16 seconds was measured at the aortic arch and used for obtaining contrast-enhanced thorax images. The CT images were acquired using iDose4 (level 3) for both groups.

**Table 1:** Showing the technical parameters and characteristic of the patient selected for the SD and LD-LV CECT Thorax groups

Variables	SD CECT Thorax (Group A)	LD-LV CECT Thorax (Group B)
Technical Parameter		
kVp	120	80
mAs	75	75
Slice thickness (millimetre)	5	5
Detector Width	64 × 0.625	64 × 0.625
FOV (millimetre)	350	350
Matrix Size	512 × 512	512 × 512
Pitch	1.00	1.00
Rotation time (seconds)	0.75	0.75
Contrast Media Volume	60 ml	40 ml
Concentration (mgI/ml)	300	300
Reconstruction algorithm	iDose^4^	iDose^4^
Post threshold delay	16 Secs	16 Secs
**Characteristics**		
Age (years)	56.12 ± 7.4	57.5 ± 9.1
Male (n=)	29	35
Female (n=)	16	10
Height	165.1 ± 1.7	164.6 ± 0.5
Weight (kilograms)	52.04 ± 12.2	52.6 ± 10.3
Body mass index	22.04 ± 2.5	23.12 ± 1.3

kVp- kilo voltage, mAs- tube current x seconds, SD- standard dose, LD-LV- Low dose low volume, CECT-Contrast enhanced computed tomography

### CT Image Analysis

CT thorax images were evaluated using predefined window width (WW) and window level (WL) settings for the lung (WW: 1300; WL: -700 HU) and mediastinal window (WW: 350; WL: 40 HU).

**Qualitative analysis**: Qualitative analysis of the CECT thorax was conducted based on the scoring criteria for CT image quality (IQ) suggested by European radiology guidelines (16). The structures evaluated in the lung included lung texture, bronchus, proximal bronchus, peripheral bronchus, adjacent vessels, clarity of lung lesion margins, and the mediastinal window, which included the trachea and surrounding soft tissue at the aortic arch level, hilar protuberances, surrounding lymph nodes, three levels of the thoracic segment of the oesophagus, the pericardium at the right ventricle level, and chest wall and muscle lesions. The lung structures were scored using a 5-point visual scoring method (17), where:

Score 1: Poor (unclear lesions, tissue structure, and heavy artifacts)
Score 2: Insufficient (fuzzy lesions, tissue structure, insufficient contrast, moderate artifacts)
Score 3: Moderate (clear lesions, partially good tissue structure, moderate contrast, slight artifacts)
Score 4: Good (clear lesions, complete tissue structure, good contrast, slight artifacts)
Score 5: Excellent (clear lesions, complete and clear tissue structure, good contrast, no artifacts)

The subjective score for overall IQ was determined by averaging the scores for the ten structures described above and rounding the result to the nearest integer. A score of 3 to 5 indicated adequate quality for diagnosis, while a score of 1 to 2 suggested inadequate quality for diagnosis. Two radiologists, each with over 10 years of experience, evaluated the CT thorax images. The readers were blinded to the dose groups.

**Quantitative analysis**: For quantitative analysis, a region of interest (ROI) of 20-40 mm^2^ was used. The Hounsfield unit (HU) of the pulmonary artery (CTPA), which represents the mean attenuation value of the main pulmonary artery (MPA), right pulmonary artery (RPA), and left pulmonary artery (LPA), was measured. The Hounsfield unit of the infraspinatus muscle (CTISM) was also measured. The standard deviation (SD) of the MPA, RPA, LPA, and infraspinatus muscle (ISM) was calculated. The contrast-to-noise ratio (CNR) was determined using the formula: CNR = (HUv - HUm)/SD: where HUv is the mean attenuation value of MPA, RPA, and LPA; HUm is the attenuation of the infraspinatus muscle; and SD is the mean of the measured ROI standard deviations, which is considered as image noise (IN).

**Radiation dose**: Radiation dose indices such as CTDIvolume (CTDIvol) and Dose-length product (DLP) were obtained from the CT console. The effective dose (ED) of CT was assessed using the following formula: ED = DLP × conversion factor (k), where k = 0.014 (18).

**Statistical analysis**: Statistical analysis was performed using SPSS (Version 20.0). The Mann-Whitney U test was used to compare qualitative image analysis between Groups A and B (iDose4). Independent t-tests were applied to assess the quantitative image analysis and radiation dose between the two groups. The Kappa statistic (poor agreement: >0.20, fair agreement: 0.20-0.40, moderate agreement: 0.40-0.60, good agreement: 0.60-0.80, excellent agreement: 0.80-1.00) was used to assess inter-observer agreement in qualitative analysis. The intra-class correlation coefficient (ICC) was used to assess inter-observer agreement for quantitative analysis, with the following scale: substantial (0.81-1.0), moderate (0.61-0.80), fair (0.41-0.60), slight (0.11-0.40), and virtually none (0.00-0.10). A p-value of <0.001 was considered statistically significant.

## Results

A total of 80 patients were included in the study. The CECT thorax SD protocol comprised 40 patients, while the LD-LV protocol included 40 patients. The patient demographic information is shown in Table 1.

**Figure 1:**
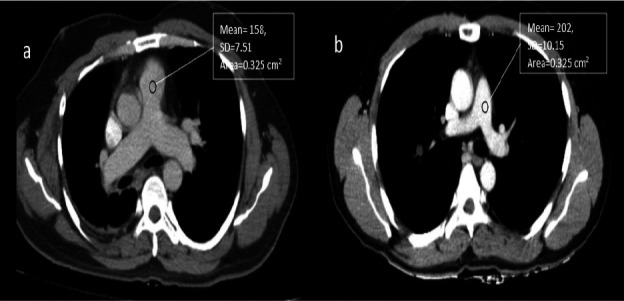
(a) Axial CECT thorax image of 45-year-old male obtained using a standard protocol (120 kVp, 60 ml, iDose^4^) (b) Axial CECT thorax image of 50-year-old male patient obtained using low dose protocol (80 kVp, 40 ml, iDose^4^)

**Qualitative analysis**: No statistically significant difference (p>0.001) was observed in the qualitative analysis between the two groups (Figure 1 a-b). The interobserver agreement (k-value) for all ten structures demonstrated excellent agreement for both Groups A and B (iDose4) (Table 2).

**Table 2:** Showing the subjective scores of the structures visualized in the lung and mediastinal window for SD and LD-LV groups

Structures	Group A											Group B											A vs B (p -value)	

Lung window	R1					R2					k	R1					R2					k	R1	R2

	5	4	3	2	1	5	4	3	2	1		5	4	3	2	1	5	4	3	2	1			
Lung texture	33	6	1	0	0	32	6	2	0	0	0.972	35	4	1	0	0	35	4	1	0	0	0.981	0.226	0.297
Bronchus	32	5	3	0	0	32	6	2	0	0	0.891	36	3	1	0	0	35	3	2	0	0	0.887	0.595	0.828
PBAV	33	6	1	0	0	32	7	1	0	0	0.869	35	2	3	0	0	36	2	2	0	0	0.919	0.717	0.845
PEBAV	31	7	2	0	0	32	6	2	0	0	0.895	34	4	2	0	0	34	3	3	0	0	0.916	0.173	0.163
CML	32	6	2	0	0	31	6	3	0	0	0.853	36	2	2	0	0	35	3	2	0	0	0.932	0.941	0.773
**Mediastinal Window**																								
TSSAA	31	7	2	0	0	32	6	2	0	0	0.937	34	4	2	0	0	35	4	1	0	0	0.876	0.325	0.637
HPSL	33	6	1	0	0	32	5	3	0	0	0.956	35	2	3	0	0	34	4	2	0	0	0.898	0.567	0.593
TLTSO	32	5	3	0	0	31	5	4	0	0	0.972	34	5	1	0	0	35	5	0	0	0	0.932	0.671	0.712
PLRV	33	5	2	0	0	33	6	1	0	0	0.947	35	4	1	0	0	34	4	2	0	0	0.945	0.319	0.416
CWMT	32	5	3	0	0	32	6	2	0	0	0.932	34	4	2	0	0	36	3	1	0	0	0.928	0.516	0.317
**OIQ**	32	6	2	0	0	31	7	2	0	0	0.953	35	3	2	0	0	35	4	1	0	0	0.877	0.253	0.593

PBAV- Proximal bronchus and adjacent vessels, PEBAV- Peripheral bronchus and adjacent vessels, CML- Clarity on margins of lesions, TSSAA- Trachea and surrounding soft tissue at the aortic arch level, HPSL- Hila protuberances and surrounding lymph nodes, TLTSO- Three levels of the thoracic segment of the oesophagus, PLRV- Pericardium at the level of the right ventricle, CWMT- Chest wall and muscle tissue, OIQ- Objective image quality, R 1-2- Reader 1 and 2

**Quantitative analysis CT_PA_ (HU)**: Statistically significant differences (p<0.001) were observed between Groups A & B (iDose4) for CTPA (Table 3; Figure 1 a-b). The ICC for CTPA indicated excellent agreement between Groups A and B (iDose4).

**Table 3:** The quantitative analysis of SD and LD-LV CECT Thorax protocol

Quantitative analysis	SD CECT Thorax (iDose^4^)			LD-LV CECT Thorax (iDose^4^)			SD vs LD-LV (iDose^4^)	
	Group A			Group B				
	**R 1**	**R 2**	**ICC**	**R 1**	**R 2**	**ICC**	**R 1**	**R 2**
**CT_PA_(HU)**	165.6±27.15	163.9±16.8	0.84	205.5±21.1	203.2±22.7	0.92	<0.001	<0.001
**CT_ISM_ (HU)**	52.2±5.9	53.6±4.1	0.87	60.1±6.5	61.5±4.5	0.95	<0.001	<0.001
**IN (sd)**	10.65±1.32	9.96±1.45	0.89	11.16±1.67	10.6±2.10	0.88	0.291	0.342
**CNR**	10.64±3.53	11.07±2.31	0.82	13.02±2.58	13.36±2.73	0.97	<0.001	<0.001

SD- Standard dose, LD-LV- Low dose low volume, CT_PA_(HU)-Hounsfield unit of pulmonary artery CT_ISM_ (HU)-Hounsfield unit of infraspinatus muscle, sd- Image noise, CNR- Contrast to noise ratio, CECT- Contrast enhanced computed tomography, R1-2 – Reader 1 and 2

**CT_ISM_ (HU)**: A statistically significant difference (p<0.001) was found between Groups A and B (iDose4) for CTISM (Table 3). The ICC for CTISM showed excellent agreement for both groups (iDose4).

**Noise (sd)**: No statistically significant difference (p>0.001) was observed in the sd between Groups A & B (iDose4) (Table 3). The ICC for SD showed excellent agreement for both groups (iDose4).

**CNR**: A statistically significant difference (p<0.001) was noted in CNR between Groups A & B (iDose4) (Table 3). The ICC for CNR indicated excellent agreement for both groups (iDose4).

**Radiation dose**: CTDIvol values for SD (9.04±3.57) and LD-LV (3.14±0.76) showed a statistically significant difference (p<0.001) between the groups, with a 65.2% reduction in radiation dose for LD-LV compared to SD. DLP values for standard (485.98±96.5) and low dose (169.22±52.05) also showed a significant difference (p<0.001) between the groups, with a 65.18% reduction in radiation dose for LD-LV compared to SD. ED values for SD (6.8±0.25) and LD-LV (2.36±0.09) showed a significant difference (p<0.001) between the groups, with a 65.3% reduction in radiation dose (Table 4).

**Table 4:** Radiation dose measurements between SD and LD-LV

Dose indices	SD CECT Thorax (iDose^4^)	LD-LV CECT Thorax (iDose^4^)	p-value
	Group A	Group B	
CTDIvol (mGy)	9.04 ± 3.57	3.14 ± 0.76	< 0.001
DLP (mGy.cm)	485.98 ± 96.5	169.22 ± 52.05	< 0.001
Effective dose (mSv)	6.8 ± 0.25	2.36±0.09	< 0.001

SD- Standard dose, LD-LV- Low dose low volume, CECT- Contrast enhanced computed tomography.

## Discussion

This study evaluated the image quality (IQ) and radiation dose (RD) of LD-LV (80 kVp; 40 ml contrast) CECT thorax in comparison to SD protocols (120 kVp; 60 ml contrast) with the iterative reconstruction algorithm (iDose4). To the best of our knowledge, this is the first study conducted with an 80 kVp and 40 ml contrast volume using the iDose4 iterative reconstruction technique for CECT thorax. Our findings suggest that the LD-LV CECT thorax protocol with iDose4 offers improved attenuation (HU), enhanced CNR, and lower RD compared to the traditional SD (120 kVp) protocol. Given the low absorption and the intrinsic contrast between vascular, interstitial structures, and surrounding lung air, low-dose scans could be highly beneficial for patients requiring CT thorax imaging. The low kVp approach would also be advantageous for patients needing follow-up CT thorax examinations to assess chest diseases.

Qualitative image analysis demonstrated excellent agreement between Group A (standard CECT thorax) and Group B (low-dose, low-volume CECT thorax). The overall image quality scores for Group B were better (not statistically significant) compared to Group A [5(87.5%), 4(7.5%), 3(5%) vs. 5(80%), 4(15%), 3(5%)]. Our findings align with a recent multicenter study by Meng et al. (19), which reported no significant difference in subjective scores between LD (100 kVp; 270 mgI/ml, 350 mgI/ml) and SD (120 kVp; 270 mgI/ml, 350 mgI/ml) groups using different iterative reconstruction algorithms from four CT vendors. Li et al. (20) also observed that the low-dose group exhibited excellent image quality, similar to the standard-dose protocol, with clear lesions and no artifacts, with diagnostic scores (≥3) for both groups (Table 5).

**Table 5:** Comparison of kVp, Iodine concentration, CTDIvol, DLP, Effective dose, reduction in radiation dose and iodine content among various studies and present study

Author (year)	This study	Meng et. al.,(19) (2019)	Li X et. al.,(20) (2022)
kVp	Low dose (80)	Low dose (100)	Low dose (100)
Iodine concentration	300 mg (I/ml)	350 (mgI/ml), 270 (mgI/ml)	270 (mgI/ml)
CTDI_Vol_ (mGy)	3.14 ± 0.76	8.3±2.9, 6.9±3.0	5.84±1.76
DLP (mGy.cm)	169.22±52.05	290.1±108.3, 241.6±104.7	199.08±57.84
Effective dose (ED)	2.36±0.09	4.1±1.5	2.79±0.81
(mSv)		3.4±1.5	
Reduction in dose (%)	ED (65.3)	ED (18, 32)	ED (36.59)
Reduction in iodine content	33.3	-	22.86
Iterative reconstruction techniques	iDose^4^	ASIR V (40%), SAFIRE(3) iDose (3), AIDR3D	ASIR V

kVp: kilo voltage, ED: Effective dose, ASIR V: Adaptive statistical iterative reconstruction, SAFIRE-Sinogram Affirmed itertative reconstruction, AIDR 3D- Adaptive iterative dose reduction

At lower photon energy levels, photoelectric interactions are more frequent. These interactions are inversely proportional to photon energy and directly proportional to the atomic number (Z) cubed. Consequently, iodine, with its higher atomic number of 53, has a larger linear attenuation coefficient as photon energy decreases (at low kVp). This enhanced photoelectric effect (PE) at reduced kVp leads to increased iodine attenuation and improved CNR (21). This also helps in reducing the amount of contrast media administered, which is beneficial for patients requiring frequent follow-up and those at risk for Contrast Induced Nephropathy (CIN). Our study showed that, with the same iodine concentration but reduced contrast media volume, the CT value of pulmonary vessels and ISM in Group B (80 kVp; iDose4) was 24.24% and 17.14% higher compared to Group A (120 kVp; iDose4). In this study, there was a 33.3% reduction in total iodine content for patients using the low-dose protocol; however, the attenuation of pulmonary vessels was higher due to enhanced PE. Li et al. (20) also reported a 22.86% reduction in iodine content with higher attenuation in the low-dose group.

Reducing the kVp can significantly lower radiation dose, much more so than adjusting the tube current. However, this may increase image noise, which can affect diagnostic image quality, especially with filtered back projection (FBP). Iterative reconstruction (IR) techniques, as the name suggests, iteratively reconstruct images to more accurately estimate mathematical assumptions, producing images with reduced noise. In our study, the noise in the LD-LV (iDose4) group was slightly higher compared to the SD (iDose4) group, but the difference was not statistically significant. Meng et al. (19) also reported that the noise values for low-dose protocols (17.9 HU, 18.1 HU) were significantly higher than those for the standard protocol (15.2, 15.5 HU) without affecting diagnostic image quality. Due to the stronger photoelectric effect at lower kVp, the CNR of contrast-enhancing structures increases (21-23). Our study also found that the CNR in LD-LV (iDose4) was greater than in SD (iDose4).

This study demonstrated a 65.3% reduction in effective dose with the LD-LV protocol. Meng et al. (19) reported an 18-32% reduction in effective dose with LD compared to SD protocols. Li et al. (20) found that the use of iterative reconstruction reduced the radiation dose by 36.59% with LD compared to SD protocols.

There are a few limitations in this study. The sample size was relatively small, and future studies with larger sample sizes and multicenter collaborations are needed to confirm the reported findings. BMI-based protocols for optimizing radiation dose and contrast media were not studied. Additionally, the diagnostic accuracy of the LD protocol in detecting and differentiating chest pathologies was not assessed.

In conclusion, the present study demonstrates that the LD (80 kVp) and LV (40 ml) CECT thorax protocol with the IR (iDose4) technique significantly reduces effective dose by 65.3% and contrast volume by 33.3%, while improving attenuation values of thoracic structures and CNR. The LD-LV protocol, in combination with iDose4, can be implemented as a routine protocol to benefit patients requiring follow-up contrast chest scans, particularly those at risk of Contrast-Induced Nephropathy (CIN), offering improved image quality and diagnostic confidence.
